# Are Socialized Services of Agricultural Green Production Conducive to the Reduction in Fertilizer Input? Empirical Evidence from Rural China

**DOI:** 10.3390/ijerph192214856

**Published:** 2022-11-11

**Authors:** Chunfang Yang, Hengyuan Zeng, Yifeng Zhang

**Affiliations:** Department of Agriculture and Forestry Economics, School of Economics and Management, Nanjing Forestry University, Nanjing 210037, China

**Keywords:** green development of agriculture, socialized services of green production, fertilizer reduction, social network

## Abstract

Reducing the use of chemical fertilizers in agricultural production is an inevitable requirement for achieving carbon neutrality and coping with global warming, and it is also an important measure for achieving green and sustainable agricultural development. Furthermore, the development of socialized services of green production provides a new approach to effectively reducing the use of fertilizers. Based on the survey data of 2202 rice growers in Jiangsu Province in 2021, this paper empirically analyzed the effects of socialized services of green production and social network on the reduction in fertilizer application by farmers. The results showed that both the socialized services of green production and social networks could significantly promote the reduction in fertilizer application by farmers. Social networks have a moderating effect between socialized services of green production and reduction in fertilizer application and can enhance the promotion of farmers’ adoption of socialized services of green production to reduce the application of fertilizers. With consideration of the potential endogenous problems of the model and the robustness test by replacing the key explanatory variables and the explained variables, all of the results were stable. Therefore, it is emphasized that the government should cultivate the main body of agricultural socialized services, improve the socialized service system of green production, and promote the green development of agriculture by service scale operation. Equally, it is necessary to strengthen the construction of rural social networks for the exchange of fertilization experience and give full play to the positive role of social networks in the reduction in fertilizer application by farmers.

## 1. Introduction

In recent years, China has made considerable progress in agricultural economic development. It has successfully fed more than 20% of the world’s population with 7% of the global cultivated land area [1], playing a vital role in safeguarding the world’s food security. However, it has also paid a huge environmental cost [2,3,4]. According to the data of the Ministry of Agriculture and Rural Affairs, agricultural non-point source pollution has surpassed industrial point source pollution to become the key source of pollution. Excessive application of chemical fertilizers is the main cause of agricultural pollution [5,6]. China’s agricultural production is highly dependent on chemical fertilizers, and there is a serious problem of overuse [7,8,9]. According to statistics, the average amount of pure chemical fertilizer per mu (1 mu = 1/15 hm^2^) of crops in China was 21.9 kg in 2015, which is much higher than the world average level of 8 kg per mu, and the average utilization rate of chemical fertilizer was only 33% [10]. The Chinese government attaches great importance to the excessive and inefficient application of agrochemicals. In order to transform agricultural production methods, promote agricultural supply-side reform, and achieve green and sustainable agricultural development, the Ministry of Agriculture and Rural Affairs issued the Action Plan for Zero Growth of Chemical Fertilizer Use by 2020 in 2015. In 2021, the “No. 1 Document” of the Central Government once again stressed the need to continuously promote the reduction in chemical fertilizers and pesticides to achieve green agricultural development, indicating that the task of reducing the application of chemical fertilizers in agricultural production is still arduous. As a response to a series of major strategies such as high-quality agricultural development and rural ecological revitalization, the reduction in chemical fertilizer has a strong positive externality, which is reflected in its ability to effectively reduce agricultural non-point source pollution, improve the sustainability of important agricultural production factors such as soil and water, and, importantly, ensure food security and achieve sustainable agricultural development.

Farmers play a central role in fertilizer reduction [11]. However, at present, 210 million farmers operate on less than 10 mu of cultivated land, and part-time, aging, and small-scale decentralized operation are still the main forms of agricultural operation in China, and the willingness and ability of small farmers to reduce fertilizer application under this form of operation are generally not high [12]. Therefore, how to scientifically and rationally guide and motivate farmers, especially small-scale farmers, to reduce fertilizer application has become the core challenge of promoting the green development of agriculture. The literature focuses on the relationship between farmland operation scale and agricultural reduction, and a large number of studies show that expanding the operation scale is conducive to the reduction in chemical fertilizers [13,14,15]. However, long-term practice shows that years of farmland transfer practice have not improved the management pattern of farmland fragmentation in China. Therefore, it is a relatively slow and difficult process to achieve chemical fertilizer reduction through land scale operation by land transfer. With the rapid development of China’s agricultural socialized service organizations, relying on its own professional and technical personnel, green means of production, low cost, and market competitive advantages, it has become an important organizational form for China to realize the transformation of agricultural production mode and promote agriculture through quality [16]. Therefore, in the new stage of agricultural development, which promotes agricultural green development in a holistic way and guides rural revitalization with green development, the service-scale operation of inducing division of labor in small farmers’ family operations through socialized services of agricultural green production provides a new way of reducing chemical fertilizer application.

It has been confirmed that agricultural socialized services play an important role in promoting farmers’ fertilizer reduction. Zhang et al. (2022) found that agricultural socialized services can significantly reduce farmers’ application of fertilizers through the moderating effects of plot size and off-farm employment of labor [17]. Zhang et al. (2022) showed through empirical research that agricultural outsourcing services can significantly promote fertilizer reduction through circuitous investment, market competition, and reputational incentives [18]. Cheng et al. (2022) analyzed the survey data of rice farmers and found that agricultural socialized services have a significant positive impact on farmers’ adoption of soil testing formulas, which can improve the probability and degree of cultivated land quality protection [19]. Cai et al. (2019) showed that the application of socialized services of green production, such as deep tillage, soil testing, and formulated fertilization in the pre-production, production, and post-production stages of food production, is the key to saving agricultural resources, improving environmental pollution and ensuring food security [20]. However, some studies have also found that agricultural socialized services have no significant impact on fertilizer application [21]. Since agricultural material distributors are the main source for farmers to purchase fertilizers and pesticides and obtain relevant information, agricultural service organizations may collude with agricultural material distributors to obtain commercial profits by inducing farmers to purchase and use fertilizers beyond the reasonable range [22]. Therefore, the participation of agricultural service organizations may aggravate the phenomenon of unreasonable fertilizer application [23].

The literature offers important insights and has reference significance for this study, but the following areas still need to be improved, which is also the contribution of this paper. Firstly, in terms of analyzing the impact of socialized services on fertilizer reduction, the existing studies mainly focus on the outsourcing of production services such as land preparation, sowing, and harvesting, while this paper further focuses on the socialized services of green production that are directly related to agricultural green production. As a brand-new form of production factors, the socialized service of agricultural green production is changing the traditional agricultural production mode, breaking the barriers of resources and environment, and guiding farmers to participate in green production [24]. The research shows that the socialized service of agricultural green production is an innovation based on the traditional agricultural socialized service, aiming at meeting the needs of high-quality agricultural development [25]. According to the specific content, it can be divided into four categories: the first category is the supply and use guidance services of green inputs such as seeds, fertilizers, pesticides, mulch films, etc.; the second category is green production technology services such as soil testing and formulated fertilization, green pest control and comprehensive utilization of straw; the third category is technical irrigation such as spray drip irrigation and green production mechanization services such as deep tillage; the fourth category is green production guarantee services such as green finance and agricultural product certification and consultation. As this paper explores whether the socialized service of agricultural green production can promote farmers’ fertilizer reduction, combined with the actual needs of farmers, the socialized service of agricultural green production is defined as socialized services directly related to agricultural green production provided to agricultural producers with environmental and ecological benefits as the main service goal, resourceful reuse of production waste and docking of recycling industries as the service purpose. The specific services selected include improved seed service, soil testing and formulated fertilization, crop cultivation and management, green pest control, water-saving irrigation and comprehensive utilization of crop straw.

Secondly, rural China is a “relational” society linked by blood and local ties. The information transfer and peer effect condensed in the social network are embedded at a rational level due to the nature of the Chinese social situation and shape the decision-making mechanism of farmers’ fertilizer application behavior. The literature focuses either on the impact of socialized services on farmers’ fertilizer reduction, with less attention paid to the role of social network, or on the impact of social networks on farmers’ fertilizer reduction, lacking discussion of socialized services. There is relatively limited research that includes socialized services and social networks in the same framework to analyze the impact of both on farmers’ fertilizer reduction applications. However, since the fertilizer market has incomplete characteristics such as non-homogeneous and concealment of quality information, farmers often make decisions based on the social network they are in. Therefore, if the social network is not included in the analysis framework when studying the influence of service outsourcing on farmers’ fertilizer reduction, this influence may be biased due to the endogenous problem of missing variables. In view of this, this paper takes 2202 rice farmers in Jiangsu Province obtained from China Land Economic Survey (CLES) in 2021 as the research object. The OLS model was used to analyze the influence of socialized services of green production and social network on farmers’ fertilizer reduction. By analyzing the interaction between socialized services of agricultural green production and social networks, the internal mechanism of realizing agricultural reduction by service-scale operation was further revealed.

The remainder of the paper is organized as follows. Section 2 provides the theoretical analysis, and Section 3 introduces the data with the variable and estimation strategy. Econometric evidence is presented in Section 4 and Section 5 and summarizes the conclusions.

## 2. Theoretical Analysis

According to the farmer behavior theory of the rational smallholder school [26], farmers, as rational economic agents pursuing maximum benefits, usually regard survival safety as the primary goal of production and operation and tend to avoid the risk of production and income reduction through intensive fertilizer input. According to externality theory, reducing the level of fertilizer application has a positive externality, the benefits of which are shared by the whole society, while the costs are borne by the farmers themselves alone; thus, farmers have insufficient incentives to reduce the application of fertilizer [27]. In addition, due to the weakness of small farmers, they face extremely high transaction costs and risks when adopting new factors or technologies, and the reduction in fertilizer application by small farmers may also lead to multiple adverse selection problems in the factor market and product market [28]. Furthermore, with the large-scale selective transfer of the rural labor force in China, the supply quantity and quality of the agricultural labor force are declining. The relative scarcity of labor resources makes the decision to replace labor with machinery a rational one, while the specificity of agricultural assets determines that agricultural socialized services are an effective way to reduce marginal costs.

According to the existing research, service-scale operation is more suitable for the smallholder production pattern under the family management system in China and is an important way to achieve agricultural reduction [18]. Agricultural socialized services embed green production technology and green production materials in the self-sufficient production process of farmers through mode and structure substitution, change the farmers’ experience of inertia mode in fertilizer application, alleviate farmers’ rigid dependence on traditional fertilizers, and realize the optimization of factor allocation for rationalized fertilizer application and fertilizer reduction. From the perspective of service providers, they will save fertilizer input based on cost considerations, build their reputation through fertilizer reduction, and obtain preferential policies [28]. In terms of factor trading, service providers have the advantage of controlling the quality of chemical inputs, and their ability to distinguish the quality of fertilizers and collect information on fertilizer efficacy is better than that of individual farmers, which can avoid the long-standing dilemma of farmers applying fertilizers through empirical judgment. Moreover, service providers have the cost advantage of bulk purchase, which can facilitate farmers’ access to and application of advanced green technologies at lower costs. Furthermore, in terms of the operation process, the level of facility, specialization, standardization, and precision of service providers can effectively avoid the nonstandard and uneven problems caused by manual fertilization, while the application of green products or technologies such as soil testing and formulated fertilization can effectively improve the efficiency of chemical fertilizer application, and then reduce the amount of chemical fertilizer application. Thus, it is believed that when agricultural socialized service organizations participate in farmers’ agricultural production, they can optimize the allocation of agricultural production factors through cost advantages and specialization advantages, overcome the weak factor endowment conditions of small farmers, and then realize a reduction in chemical fertilizer application. Accordingly, the research hypothesis is proposed.

**Hypothesis** **1.** *Farmers’ adoption of socialized services of agricultural green production can promote the reduction in chemical fertilizer application*.

Economic behavior is embedded in social networks, while social networks based on trust and reputation maintain the existence of economic relations and systems [29]. In rural China, which is typically characterized by “local” and “kinship”, the influence of social networks on the production and management or life decisions of farmers’ households is particularly important. As “social people”, farmers’ production decisions are influenced not only by their own internal factors but also by the social network in which they are embedded [30]. Specifically, social networks affect farmers’ fertilizer reduction through, among others, the following three mechanisms: first, the information acquisition mechanism. Studies have shown that social networks are an important channel for farmers to obtain information on agricultural technologies [31,32]. With the help of social networks, farmers can effectively obtain information on green production technologies, reduce information asymmetry, reduce transaction costs in the process of green production technology adoption, and thus promote the rational application of fertilizers. The unique relational social network in rural China makes information transmission and collective communication and decision-making among farmers the main channel of agricultural technology diffusion [33]. Second, the interactive learning mechanism. Farmers embedded in a social network are influenced by other members within the social network and exhibit a “peer effect” [34]. Goyal et al. (2007) found that relying on “model households” to disseminate technical information can reduce the time and cost for surrounding farmers to obtain technical information, thus promoting technology adoption [35]. Carter et al. (2016) also found that social networks help farmers learn from each other and promote their adoption of green production technologies [36]. Gerba et al. (2018) and Aida (2018) stated that most farmers rely on social learning to adopt agricultural technologies and acquire knowledge mainly through social networks in the form of communication, observation, and participation in socio-cultural activities [37,38]. Third, the helper support mechanism. Bian et al. (2021) pointed out that social networks have a human helper function [39]. Good social norms and mutual trust in social networks can promote cooperation and reciprocity among farmers, and richer social networks often mean that smallholders have access to financial, technical, and labor support for the adoption of technical services for agricultural production.

At present, China’s fertilizer market is characterized by incomplete information on fertilizer elements markets such as non-homogeneity, the confusing scenario of the agricultural market, and the concealment of chemical quality information. From the perspective of agricultural reduction, the characteristics of social networks such as information transfer, human help, and reciprocal learning can effectively compensate for the deficiencies of China’s fertilizer factor market. In addition, the extension service of agricultural green production technology in China is not perfect, and social networks can compensate for the mismatch of technical information to a certain extent, thus improving the adoption of socialized services of green production by rice farmers to promote the reduction in chemical fertilizer application. Furthermore, participation in agricultural socialized service organizations can significantly broaden social networks. It can be seen that the interaction between socialized services of agricultural green production and social networks may be an important mechanism to encourage rice farmers to reduce fertilizer application. Therefore, the following research hypotheses are proposed.

**Hypothesis** **2a.** *Social networks are conducive to promoting the reduction in fertilizer application by farmers.*

**Hypothesis** **2b.** *Social networks have a moderating effect between socialized services of agricultural green production and fertilizer reduction application, and the richer the farmer’s social network, the greater the influence of socialized services of agricultural green production on fertilizer reduction application.*

## 3. Materials and Methods

### 3.1. Data Sources

China is a large rice-producing country, yet excessive inputs of chemical fertilizers in rice cultivation have exacerbated and continue to exacerbate soil degradation, greenhouse gas emissions, and groundwater pollution [40]. Therefore, this paper focuses on the use of chemical fertilizers in rice production. Jiangsu Province was selected as the sample province for the following reasons: on the one hand, it is the key area for popularizing agricultural green production technology in China, and it has good representativeness and reference value for studying farmers’ green production of rice; on the other hand, it is an important rice-producing area in China. In 2020, the sown area and yield of rice accounted for 7.3% and 9.3% of the whole country, respectively. However, excessive and inefficient application of chemical fertilizers in the production process is very prominent [41]. Therefore, this paper mainly investigates the adoption of socialized services of agricultural green production and the reduction in fertilizer application by rice growers in Jiangsu Province, and the research is representative.

**Figure 1:**
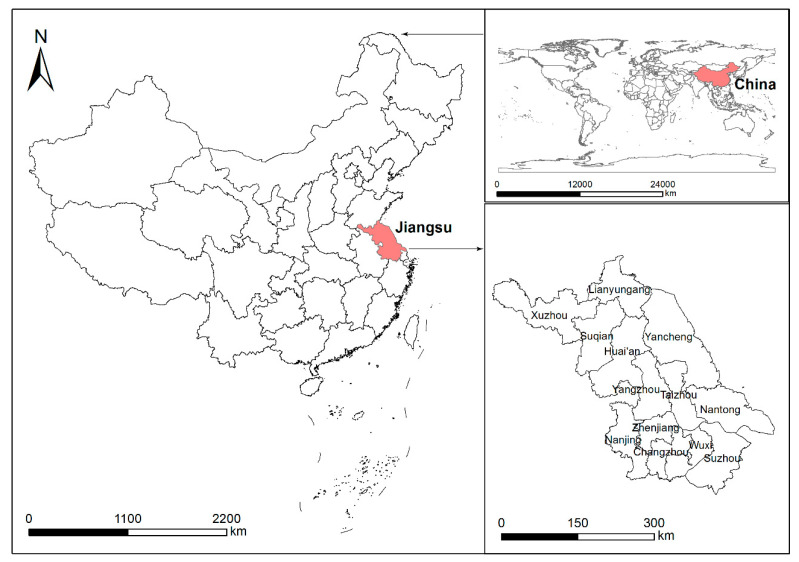
Distribution map of the sample area.

The data sources used for analysis are the China Land Economic Survey (CLES), which was established by the Department of Humanities and Social Sciences of Nanjing Agricultural University in 2020 and assisted by the Jin Shanbao Institute of Modern Agricultural in Jiangsu Province. The data used in this paper are from a tracking survey of the project in 2021, and the questionnaire covers basic household information, land market, agricultural production, ecological environment, and other aspects, and provides data support for the empirical study of this paper. The sampling method of probability proportional to size (PPS) was adopted in the survey. A total of 26 counties were randomly selected from thirteen prefecture-level cities of Jiangsu Province (Figure 1). Two sample townships were randomly chosen from each county, and one administrative village was randomly selected from each township. Finally, the researchers randomly selected fifty households in every village. The sample comprised 52 villages and 2420 households in 2021. After processing the abnormal and missing values of variables, 2202 valid samples were finally retained.

### 3.2. Variable Selection and Statistical Description

Chemical fertilizer is the main chemical input in agricultural production, so this paper selected the actual fertilizer application amount of farmers’ rice production as the explained variable. It was measured by the actual fertilizer input per mu (jin/mu) of farmers. Considering the significant difference in the amount of fertilizer application among different crop varieties, this paper only investigated the chemical fertilizer input in the process of rice planting.

The core explanatory variable of this paper is the degree to which farmers adopt socialized services of agricultural green production. Based on the consideration of fertilizer reduction and efficiency increase, and referring to the research of Willy et al. (2013) [42] and Li et al. (2021) [24], this paper selected six types of socialized services of agricultural green production, which cover the main processes of agricultural production and are closely related to the reduction in chemical fertilizer application, including improved seed service, soil testing, and formulated fertilization, crop cultivation and management, green pest control, water-saving irrigation, and comprehensive utilization of crop straw. According to the number of types of socialized services actually adopted by farmers, there are 7 kinds: “not adopted” = 0, “adopted 1 service” = 1, “adopted 2 services” = 2, “adopted 3 services” = 3, “adopted 4 services” = 4, “adopted 5 services” = 5, and “adopted 6 services” = 6.

Theoretical analysis shows that social networks play an important regulating role between socialized services of green production and fertilizer reduction application by farmers. Drawing on the research of Yang et al. (2019) [43], this paper selected the expenditure on human gifts as the proxy variable of social networks. Rural China is a typical human society, which follows the traditional social approach of “courtesy demands reciprocity”. Based on the household’s expenditure on human gifts, we can not only accurately determine the size of the household’s social network but also objectively identify the size of the farmer’s access to resources such as knowledge and technical information based on these networks.

In order to avoid the model estimation bias caused by missing variables, referring to the research of Wossen et al. (2017) [44] and Moslem et al. (2020) [45], this paper selected the personal characteristics of the head of household, that is, gender, age, education level, health status, agricultural technology training, and risk preference; family characteristics, that is, the number of agricultural labor force and total household income; agricultural production and operation characteristics, that is, planting insurance, rice planting area, plot size, field traffic conditions, and soil fertility as control variables. The descriptive statistics of all variables are shown in Table 1.

### 3.3. Model Specification and Estimation Methods

#### 3.3.1. The Benchmark Regression Model: Ordinary Least Squares Model

In order to examine the influence of socialized services of green production on farmers’ fertilizer reduction application, this paper used ordinary least squares estimation to build a model as follows:

(1) $$\ln Fertilizer_{i}=\beta_{0}+\beta_{1}Service_{i}+\beta_{2}\ln Social_{i}+\beta_{3}C_{i}+\varepsilon_{i}$$

where *Fertilizer_i_* denotes the average fertilizer application per mu (jin/mu) of rice of the *i* farmer, ln*Fertilizer_i_* is the logarithm of fertilizer application amount; *Service_i_* refers to the number of socialized services of green production adopted by the *i* farmer; *C_i_* is the control variable, including individual characteristics, family characteristics, agricultural production and operation characteristics, etc. *β*_0_, *β*_1_, *β*_2_, and *β*_3_ are the coefficients to be estimated, and *ε_i_* is the random disturbance term.

#### 3.3.2. Moderating Effect Model

Based on the above theoretical analysis, it can be seen that social networks may play a moderating role in the effect of farmers’ adoption of socialized services of green production on fertilizer reduction. To test the moderating effect, this paper establishes the following moderating effect model:

(2) $$\ln Fertilizer_{i}=\beta_{0}+\beta_{1}Service_{i}+\beta_{2}\ln Social_{i}+\beta_{3}Service_{i}\times \ln Social_{i}+\beta_{4}C_{i}+\varepsilon_{i}$$

In Equation (2), *Service_i_* × ln*Social_i_* denotes the interaction term between the socialized services of green production and the social network; *C_i_* is the control variable, including individual characteristics, family characteristics, agricultural production and operation characteristics, etc. *β*_0_, *β*_1_, *β*_2_, *β*_3_ and *β*_4_ are the coefficients to be estimated, and *ε_i_* is the random disturbance term.

## 4. Results and Discussion

### 4.1. Basic Estimate Discussion

For this paper, Stata16.0 software was used to estimate the model. Considering that there may be multicollinearity among variables, we first used the variance inflation factor (VIF) to test the multicollinearity of all variables. The results show that the VIF of each variable is less than 2 and the mean value is 1.27, indicating that the model does not have the problem of multicollinearity. The estimated results of the ordinary least squares method are reported in Table 2. First, the impact of socialized services of agricultural green production on farmers’ fertilizer application was examined, and the estimated results are presented in model (1); second, the social network was included in the regression to obtain model (2) to examine the impact of socialized services of agricultural green production and social networks on farmers’ fertilizer application in an integrated manner; finally, the interaction term between socialized services of agricultural green production and social networks was introduced in the model to examine the influential mechanism of social networks in socialized services of agricultural green production on farmers’ fertilizer reduction application, and the estimated results are presented in model (3).

The results of model (1) show that the estimated coefficient of socialized services of agricultural green production is significantly negative at the 1% statistical level, which indicates that socialized services of agricultural green production have a significant negative effect on chemical fertilizer application. In other words, under otherwise the same conditions, the improvement of farmers’ adoption level of socialized services of agricultural green production can significantly reduce the amount of chemical fertilizer application. Hypothesis 1 is verified. Compared with small farmers, agricultural socialized service organizations have a strong ability to collect and identify fertilizer efficiency information, so as to overcome the dilemma that farmers usually judge the amount of chemicals input based on experience. On the other hand, agricultural socialized service organizations are equipped with advanced agricultural reduction technologies and tools, which can improve the efficiency of fertilizer utilization by farmers in a specialized and intensive manner, thus realizing fertilizer reduction.

The estimated results of model (2) show that the social network is significant at the 5% statistical level, and the estimation coefficient is negative, indicating that social networks can promote the reduction in fertilizer application, which is consistent with the research conclusion of Lv et al. (2021) [46]. Hypothesis 2a was verified. Under the condition of asymmetric information in the fertilizer market, farmers’ fertilizer application decisions deviate from the optimal decision, which leads to the loss of production efficiency. As an important channel for information acquisition in acquaintance societies, social networks can break the information barrier and reduce the information asymmetry in agriculture, reduce the cost of information search, and provide conditions for farmers to “learn by watching” and “learn by doing” in the process of technology adoption so that farmers can make optimal fertilizer application choices under the condition of information asymmetry.

The estimated results of model (3) show that the interaction between socialized services of agricultural green production and social networks was significantly negative at a 5% statistical level, indicating that there was a complementary relationship between the two in promoting the reduction in chemical fertilizer application by rice-growing farmers. Complemented with a social network, the influence of socialized services of agricultural green production on reducing chemical fertilizer application per mu of rice by farmers was further developed, thus verifying hypothesis 2b. Under a certain level of adoption of social services of green production by farmers, social networks can effectively promote chemical fertilizer application reduction. In other words, on the one hand, social networks can effectively compensate for the insufficient adoption of socialized services of agricultural green production by farmers. At present, China’s agricultural technology extension services are not sound, and farmers mainly use new technologies by observing, learning, and interacting with people around them, which compensates for the mismatch of technical information to a certain extent. In addition, social networks can enrich the agricultural knowledge of rice farmers, and thus increase their awareness of socialized services of green production and rational fertilizer application to promote the reduction in chemical fertilizer application. On the other hand, when the quality of social networks is low, the socialized services of agricultural green production obtained by farmers can also promote the reduction in fertilizer application, and the purchase of social services of green production by farmers can also broaden the social network of farmers to a certain extent. Therefore, the complementary effect between socialized services of green production and social networks may be an important mechanism to encourage rice farmers to reduce fertilizer application.

In terms of control variables, health status, agricultural technical training, planting insurance, and field traffic conditions have a significant impact on farmers’ chemical fertilizer application. The better the health status of the head of household, the more likely farmers are to choose to use excessive chemical fertilizer, which may be due to the substitution effect between labor force and chemical fertilizer. A physically healthy labor force can readily take up employment, and farmers tend to use excessive chemical fertilizer input to realize the transfer of labor force. Agricultural technical training has a significant negative impact on the average fertilizer application per mu of rice, which indicates that farmers who have received agricultural technical training have a higher awareness of the input efficiency of agricultural chemicals than ordinary farmers and can then promote the reduction in fertilizer application. The purchase of planting insurance has a significant positive effect on fertilizer application. This suggests that the negative impact of moral hazard on China’s “low security, wide coverage” agricultural insurance system cannot be ignored, and the incentives of different government financial support policies may be inconsistent, which makes it difficult to maximize the policy effect. Field traffic conditions significantly and positively affected fertilizer application, i.e., the greater the distance from hardened concrete roads, the more farmers tended to over-apply fertilizer. The possible reason for this is that since the management of plots with inconvenient traffic needs to consume more costs, farmers tend to increase the amount of fertilizer applied in a single application in order to reduce the frequency of management, thus leading to excessive application of fertilizer [47].

### 4.2. Endogeneity

The issue of endogeneity is an important challenge in studying farmers’ behavior in decision-making and its influencing factors. In order to overcome the possible endogenous problems of the model, this paper will further introduce an instrumental variable to explore the impact of socialized services of agricultural green production on farmers’ fertilizer application, so as to verify the stability of the above empirical results. The instrumental variable needs to be highly correlated with endogenous variables (socialized services of green production) but does not directly affect the explained variable (fertilizer application amount). Referring to the practice of Liang et al. (2020) [48], the terrain is regarded as an instrumental variable for socialized services of green production. The indicator was measured by asking “What is the terrain of the largest piece of land in your home?”. If farmers chose flat land, the value was set to 1; otherwise, it was set to 0. Terrain significantly affects the development of the agricultural socialized service market, which is the main reason for the regional characteristics of the development of agricultural socialized services, especially mechanical services in China [49]. Hu et al. (2018) also confirmed that terrain significantly affects farmers’ adoption of outsourcing services [50]. However, the terrain will not directly affect farmers’ fertilizer input but indirectly affects farmers’ fertilizer input through the availability and price of agricultural socialized services.

Table 3 presents the estimated results of the two instrumental variable methods. The IV estimated results of model (1) show that the influence of terrain on farmers’ fertilizer application is consistent with the benchmark regression in direction and significance level, which shows that the conclusion is still valid after overcoming the potential endogenous problems of the model. The results of the Durbin–Wu–Hausman (DWH) test passed the 1% significance level, rejecting the original hypothesis that the socialized services of green production are an exogenous variable. The F-statistic of the weak instrumental variable test is 47.19, which is much larger than the critical value of 10 for weak instrumental variables, and the original hypothesis of the existence of weak instrumental variables can be rejected. Furthermore, the limited information maximum likelihood method (LIML), which is less sensitive to weak instrumental variables, is used for estimation. The results of model (2) show that the coefficient estimation and significance level of LIML are consistent with IV estimation, which proves that there is no weak instrumental variable, indicating that the instrumental variable selected in this paper is effective.

### 4.3. Robustness Test

In order to test the robustness of benchmark regression results, this paper uses the method of replacing core explanatory variables and explained variables for robustness testing, and the results are shown in Table 4. Firstly, the independent variable, the degree of adoption of socialized services of green production, is replaced by the question of whether to adopt socialized services of green production for the robustness test (if farmers have adopted at least one of the socialized services of green production specified in this paper, the value is 1; otherwise, it is 0). The results of model (1) show that the question of whether to adopt socialized services of green production significantly reduces the fertilizer application of rice farmers at the statistical level of 1%, which is consistent with the previous benchmark regression conclusion. Secondly, given that some farmers in the sample have purchased and used agricultural machinery independently, does it mean that farmers can achieve fertilizer application reduction by using their own machinery to carry out mechanical operations in the agricultural production? If so, it may overestimate the impact of socialized services of green production on agricultural reduction. Based on this, this paper takes the number of farm machines owned by farmers as the core explanatory variable to re-estimate the model. The results of model (2) showed that the amount of farm machines owned by farmers did not have a significant negative impact on fertilizer application, which indicated that the reduction in chemical fertilizer application still depended on the use of socialized services of agricultural green production.

In addition, this paper chose the average fertilizer input cost per mu of rice as the alternative variable for the average fertilizer application amount per mu for robustness testing, and the results are shown in model (3). The socialized services of agricultural green production have a significant negative impact on farmers’ fertilizer input cost, which promotes the reduction in fertilizer application. At the same time, the estimated results of other variables are consistent with the above. Obviously, the above research results are still valid after taking the input cost of chemical fertilizer as the substitute variable of chemical fertilizer application amount. At this point, the core assumptions of this paper have been further verified.

### 4.4. Extensive Analysis

For this paper, we further analyzed the influence of socialized services of agricultural green production on farmers’ fertilizer application amounts with different social network endowments. Since there is no uniform standard for dividing the size of a social network, this paper divides farmers by the mean value of the social network and defines farmers with social network levels greater than 7.702 as higher social network-level farmers (i.e., above average) and vice versa as lower social network-level farmers (i.e., below average). Comparing the estimated results of model (1) and model (2) in Table 5, we can see that the socialized services of agricultural green production have a significant effect on the fertilizer reduction application of farmers at different social network levels, but the socialized services of agricultural green production have a more significant effect on the fertilizer reduction in farmers at high social network levels than those at low social network levels, and this empirical result further verifies hypothesis 2b of this paper. It can be seen that farmers broaden their social network through favorable contact, and the expansion of a social network can achieve sufficient exchange of information on socialized services of agricultural green production, which in turn promotes their adoption of these services to achieve fertilizer application reduction.

## 5. Conclusions and Policy Implications

### 5.1. Conclusions

To explain the logical mechanism of socialized services of agricultural green production promoting farmers’ fertilizer reduction, this paper used the survey data of 2202 rice farmers in Jiangsu Province and the OLS model to empirically test the impact of these services on fertilizer reduction. By constructing the moderating effect model, the moderating effect of social networks was tested. The results show that the adoption of socialized services of agricultural green production by farmers can significantly reduce the amount of chemical fertilizer application; social networks also make a significant contribution to fertilizer reduction. The moderating effect analysis shows that social networks can significantly enhance the influence of farmers’ adoption of socialized services of agricultural green production on the reduction in fertilizer application. In addition, according to the average value of social networks, farmers are divided into low social network-level farmers and high social network-level farmers. It was found that the adoption of socialized services of agricultural green production had a more significant fertilizer reduction effect on high social network-level farmers than low social network-level farmers, which further confirmed the research hypothesis (hypothesis 2b) of this paper. Therefore, it can be judged that, against the realistic background of the basic pattern of decentralized management of small farmers in China, it is difficult to change fundamentally in the short term, and it is of great significance to realize fertilizer reduction by outsourcing services. At the same time, the expansion of farmers’ social network can compensate for the imperfections of China’s socialized service market and fertilizer market, and its role in China’s agricultural green development cannot be ignored. Our research conclusions are consistent with those of previous researchers [16,17], who also believed that socialized service can promote farmers’ fertilizer reduction, but contrary to the conclusions of other studies [7,51], which suggested that with the commercialization of service organizations, service providers may display opportunistic behavior, such as collusion with agricultural material distributors; thus, service outsourcing not only does not help to reduce inputs but also leads to abuse of inputs. These gaps may be due to differences in situational factors across studies and the fact that the sampling of existing studies is mostly limited to a particular crop, time, or location, which weakens the generalizability of the findings. In practice, many situational factors often lead to different correlations between variables, and although service outsourcing contributes to fertilizer reduction, the moderating variables and implicit mechanisms between them cannot be ignored.

### 5.2. Policy Implications

Based on the above research conclusions, this paper puts forward the following policy suggestions: first, cultivate socialized service organizations and consider socialized services as an important way to promote fertilizer reduction and realize the green development of agriculture. The green development of agriculture should focus on improving the socialized service system of green production, guiding and supporting various socialized service entities to better provide farmers with services related to the whole industrial chain of green production. Second, the development of socialized services depends on sufficient market capacity, so farmers should be encouraged to carry out contiguous planting to realize the transformation from decentralization and fragmentation to large-scale operation and realize the reduction effect of chemical fertilizer application. In addition, farmers usually apply fertilizer based on empirical judgment and past habits, so it is necessary to strengthen the relevant technical training for farmers and improve their scientific awareness of the rational use of chemical fertilizers and environmental protection through training. Third, strengthen rural social networks for the exchange of fertilization experience. The high information symmetry of acquaintance society is conducive to improving the utilization efficiency of information transmission and transformation. Local governments should pay full attention to the important role of rural social networks in farmers’ fertilizer reduction. They can create conditions for farmers’ interaction, broaden information exchange channels, and expand social networks by setting up production mutual aid groups, establishing village-level WeChat groups, and organizing indoor and outdoor forums and field observation.

Due to limited research resources and objective conditions, this paper has the following shortcomings: first, the data of this empirical analysis come from the survey of rice farmers in Jiangsu Province, and the representativeness of the sample may be relatively limited. For this reason, subsequent research could improve the representativeness of sample data through cross-regional research and carry out comparative research on geospatial dimensions. Second, when analyzing the influence of socialized services of green production on farmers’ fertilizer reduction, we failed to compare the effect of different socialized services on farmers’ fertilizer reduction. It is suggested that further discussion should be carried out in future research to accurately locate the key links of socialized services of agricultural green production to promote farmers’ fertilizer reduction and put forward targeted measures.

## Figures and Tables

**Table 1:** Definitions and descriptive statistics of the survey data.

Variable	Definition	Mean	SD
Fertilizer application amount	Logarithm of farmers’ actual fertilizer input (jin/mu)	2.520	1.788
Socialized services of green production	Actual number of socialized services adopted by farmers, ranging from 0 to 6	2.009	1.187
Social network	Take the logarithm of the payment of human gifts (yuan)	7.702	2.624
Gender	Gender of the head of household: 1 = male, 0 = female	0.926	0.262
Age	Actual age of the head of household (years)	63.396	10.604
Education level	Actual years of education of the head of household (years)	7.312	3.649
Health status	Health level of the head of household, between 1 and 5: 1 = very unhealthy, 2 = unhealthy, 3 = average, 4 = healthy, 5 = very healthy	4.012	1.095
Agricultural technology training	Whether the household head has been trained in agricultural technology: 1 = yes, 0 = no	0.315	0.465
Risk preference	Degree of risk preference of the head of household, ranging from 1 to 3: 1 = risk preference, 2 = risk neutrality, 3 = risk aversion	2.719	0.554
Family agricultural labor	Number of family agricultural laborers	1.432	1.003
Total household income	Logarithm of total household income (yuan)	2.024	3.969
Planting insurance	Logarithm of planting insurance (yuan)	1.425	2.374
Plot size	Largest piece of land in the household (mu)	2.814	6.143
Rice planting area	Total rice planting area (mu)	12.915	88.664
Field traffic conditions	Distance from the largest plot to the nearest hardened cement road (miles)	1.294	14.932
Soil fertility	Soil fertility: 1 = poor, 2 = medium, 3 = good	2.476	0.620

**Table 2:** Estimated results of the impact of socialized services of agricultural green production and social networks on farmers’ chemical fertilizer application.

Variable	Fertilizer Application Amount (jin/mu)		
	(1)	(2)	(3)
Socialized service of green production	−0.089 *** (0.031)	−0.084 *** (0.031)	−0.228 ** (0.091)
Social network	-	−0.010 ** (0.007)	−0.012 * (0.008)
Socialized service of green production × Social network	-	-	−0.016 ** (0.010)
Gender	−0.062 (0.072)	−0.077 (0.071)	−0.076 (0.072)
Age	−0.001 (0.001)	−0.002 (0.001)	−0.002 (0.001)
Education level	0.003 (0.006)	0.004 (0.006)	0.005 (0.006)
Health status	0.040 ** (0.015)	0.038 ** (0.014)	0.035 ** (0.014)
Agricultural technical training	−0.062 * (0.044)	−0.080 * (0.044)	−0.071 * (0.044)
Risk preference	0.0190 (0.035)	0.022 (0.036)	0.024 (0.036)
Family agricultural labor	−0.021 (0.023)	−0.015 (0.031)	−0.012 (0.031)
Planting insurance	0.022 *** (0.008)	0.021 *** (0.008)	0.021 *** (0.008)
Total household income	−0.005 (0.008)	−0.005 (0.008)	−0.006 (0.008)
Plot size	−0.004 (0.005)	−0.004 (0.005)	−0.004 (0.005)
Planting insurance	−0.000 (0.000)	−0.000 (0.000)	−0.000 (0.000)
Field traffic conditions	0.016 *** (0.004)	0.016 *** (0.004)	0.016 *** (0.004)
Soil fertility	−0.025 (0.031)	−0.027 (0.031)	−0.027 (0.031)
Constant	5.192 *** (0.193)	5.260 *** (0.206)	5.290 *** (0.209)
N	2202	2202	2202
R2	0.044	0.047	0.053

Note: ***, **, and * denote significance at 1%, 5%, and 10% level, respectively; robust standard errors are presented in parentheses.

**Table 3:** Socialized services of green production and fertilizer reduction: endogenous test.

Variable	IV-2SLS (1)	LIML (2)
Terrain	−0.107 *** (0.056)	−0.107 *** (0.056)
Control variable	YES	YES
Constant	5.501 *** (0.216)	5.501 *** (0.216)
Durbin–Wu–Hausman test	14.540 ***	14.540 ***
Weak instrumental variable test: F value	47.19	47.19
R^2^	0.049	0.049

Note: *** denote significance at 1%; robust standard errors are presented in parentheses; due to limited space, the regression results of the control variables are not reported.

**Table 4:** Socialized services of green production and fertilizer reduction: robustness test.

Variable	Fertilizer Application Amount (jin/mu)		Fertilizer Input Cost (yuan/mu)
	(1)	(2)	(3)
Whether to adopt socialized services of green production	−0.213 *** (0.060)	-	-
Number of farm machines owned by farmers	-	0.001 (0.014)	-
Socialized services of green production	-	-	−0.103 *** (0.015)
Social network	-	-	−0.072 * (0.004)
Socialized services of green production×Social network	-	-	−0.090 * (0.006)
Control variable	YES	YES	YES
Constant	5.281 *** (0.251)	4.423 *** (0.315)	5.017 *** (0.154)
N	2202	2202	2202
R^2^	0.047	0.041	0.062

Note: ***, * denote significance at 1%, and 10% level, respectively; robust standard errors are presented in parentheses; due to limited space, the regression results of the control variables are not reported.

**Table 5:** Influence of socialized services of green production on farmers’ fertilizer use with different social network levels: extended analysis.

Variable	Social Network	
	Below Average (1)	Above Average (2)
Social services of green production	−0.074 * (0.016)	−0.235 ** (0.051)
Control variable	YES	YES
Constant	4.304 ***	5.283 ***
N	701	1501
R^2^	0.071	0.125

Note: ***, **, and * denote significance at 1%, 5%, and 10% level, respectively; robust standard errors are presented in parentheses; due to limited space, the regression results of the control variables are not reported.

## Data Availability

The data underlying the results presented in the study are all available. The data presented in this study are available on request from the corresponding author. The data are not publicly available due to privacy.
